# Finding long tandem repeats in long noisy reads

**DOI:** 10.1093/bioinformatics/btaa865

**Published:** 2020-10-08

**Authors:** Shinichi Morishita, Kazuki Ichikawa, Eugene W Myers

**Affiliations:** Department of Computational Biology and Medical Sciences, Graduate School of Frontier Sciences, The University of Tokyo, Chiba 277-8562, Japan; Department of Computational Biology and Medical Sciences, Graduate School of Frontier Sciences, The University of Tokyo, Chiba 277-8562, Japan; Max Planck Institute of Molecular Cell Biology and Genetics, Dresden, Saxony 01307, Germany; Center for Systems Biology Dresden, Dresden, Saxony 01307, Germany

## Abstract

**Motivation:**

Long tandem repeat expansions of more than 1000 nt have been suggested to be associated with diseases, but remain largely unexplored in individual human genomes because read lengths have been too short. However, new long-read sequencing technologies can produce single reads of 10 000 nt or more that can span such repeat expansions, although these long reads have high error rates, of 10–20%, which complicates the detection of repetitive elements. Moreover, most traditional algorithms for finding tandem repeats are designed to find short tandem repeats (<1000 nt) and cannot effectively handle the high error rate of long reads in a reasonable amount of time.

**Results:**

Here, we report an efficient algorithm for solving this problem that takes advantage of the length of the repeat. Namely, a long tandem repeat has hundreds or thousands of approximate copies of the repeated unit, so despite the error rate, many short *k*-mers will be error-free in many copies of the unit. We exploited this characteristic to develop a method for first estimating regions that could contain a tandem repeat, by analyzing the *k*-mer frequency distributions of fixed-size windows across the target read, followed by an algorithm that assembles the *k*-mers of a putative region into the consensus repeat unit by greedily traversing a de Bruijn graph. Experimental results indicated that the proposed algorithm largely outperformed Tandem Repeats Finder, a widely used program for finding tandem repeats, in terms of sensitivity.

**Availability and implementation:**

https://github.com/morisUtokyo/mTR.

## 1 Introduction

Tandem repeats are prevalent in genomes, and expansions of short tandem repeats (STRs), repetitive elements of 2–6 nt in length, are known to be associated with several brain diseases (Mirkin, 2007). For example, the triplet repeat ${\left(\text{CAG}\right)}_{n}$ in the coding region of the Huntington gene is short (n<28) in normal samples, but it becomes long (n>40) in patients with Huntington’s disease (MacDonald *et al.*, 1993). Short-read sequencing technologies that produce reads of 150 nt are able to identify the strings of these relatively short STR expansions. However, longer STRs of 1000 nt or more remain largely unexplored.

Despite this sequencing difficulty, a number of long STRs have been reported to be associated with brain diseases using a restriction enzymes strategy. Specifically, an STR can be enriched if it does not contain the recognition sites of a set of restriction enzymes that digest genomic regions other than the focal STR. This technical approach has uncovered a number of STR expansions associated with brain diseases in exons, introns and untranslated regions (UTRs). For example, a (CGG) repeat in the 5′-UTR is correlated with fragile-X syndrome (Kremer *et al.*, 1991; Sherman *et al.*, 1985; Verkerk *et al.*, 1991) and with neuronal intranuclear inclusion disease and oculopharyngodistal myopathy (Ishiura *et al.*, 2019), a (CTG) repeat in the 3′-UTR is associated with myotonic dystrophy type 1 (DM1) (Brook *et al.*, 1992; Mahadevan *et al.*, 1992), a (CCTG) repeat in an intron causes myotonic dystrophy type 2 (DM2) (Liquori *et al.*, 2001), a (GGGGCC) repeat in an intron is correlated with amyotrophic lateral sclerosis/frontotemporal dementia (ALS/FTD) (DeJesus-Hernandez *et al.*, 2011; Orr, 2011; Renton *et al.*, 2011). Although this restriction enzyme approach is cost-efficient, it requires prior knowledge of the details of the focal STRs and is therefore not capable of searching the entire genome for *de novo* STRs.

The recent advances in sequencing long DNA fragments of more than 10K nt has made it possible to capture many novel tandem repeats in genomes, including STRs. Loomis *et al.* first demonstrated the usefulness of Pacific Bioscience’s single-molecule real-time (SMRT) long-read sequencing technology by reading an instance of the fragile X locus with 750 tandem copes of the (CGG) unit (Loomis *et al.*, 2013), a region with 100% GC-content. Recently, Ishiura *et al.* uncovered novel intronic (TTTCA) and (TTTTA) tandem repeat expansions of ∼5K nt for benign adult familial myoclonic epilepsy using both Nanopore and PacBio sequencers, and showed that their repeat length correlates with the onset age of epilepsy (Ishiura *et al.*, 2018).

Another relevant application of long-read sequencing is the *de novo* assembly of genomes of many species, including humans. Chaisson *et al.* attempted to close gaps in the human genome using SMRT sequencing and found that many of the gaps were filled with long tandem repeats, including centromeric tandem repeats (Chaisson *et al.*, 2015). Although sequencing human centromeres with thousands of alpha-satellite monomers and their higher order structures is still a daunting challenge, a recent study did determine the centromere sequence of chromosome Y by sequencing several BAC clones covering the region with Nanopore reads (Jain *et al.*, 2018).

**Fig. 1.**
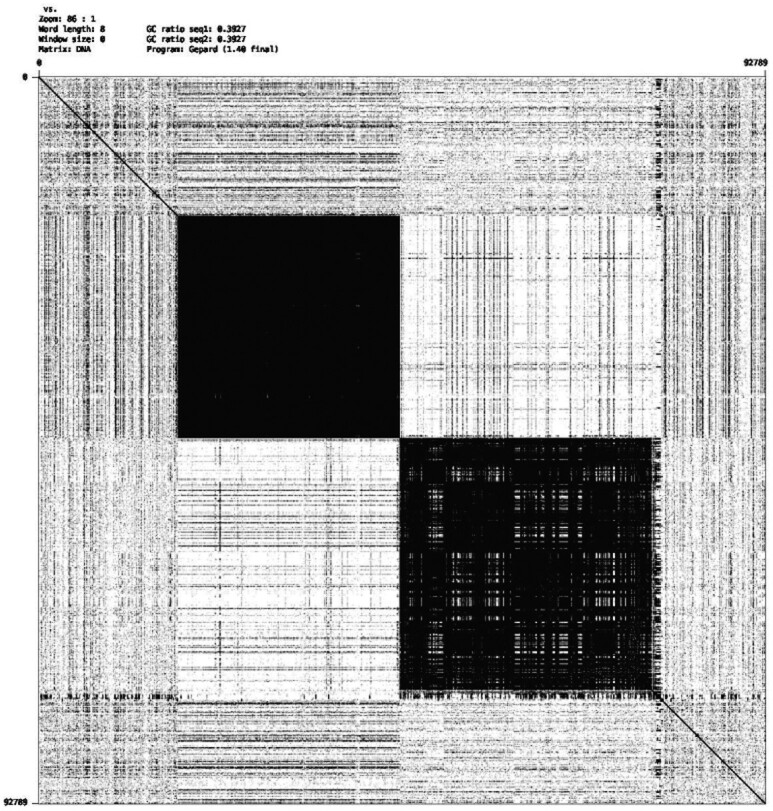
A self dot plot of a ∼92K nt Nanopore read with two different types of neighboring long tandem repeats. The upper left tandem repeat has 1124 copies of a 25 nt unit and matches its corresponding perfect tandem repeat at an identity of ∼87.8%. The lower right has 1216 copies of a 27 nt unit and matches at ∼69.7%, though its plaid pattern may imply the presence of several shorter tandem repeats

In another project (Yoshimura *et al.*, 2019), strain VC2010 of *Caenorhabditis elegans* was sequenced with long-read technologies in an attempt to produce a gapless reconstruction, the motivation being in part to rectify the mounting evidence that the original N2-genome, originally reported to be gap-free, is missing sequence segments. Widely used genome assemblers such as Canu (Koren *et al.*, 2017), FALCON (Chin *et al.*, 2016) and miniasm (Li, 2016) output assemblies of PacBio reads with 76–202 gaps, all but 5 of which could be closed by semi-manual means using the combined evidence of all the assemblies. Ultra-long Nanopore reads were then generated, and these closed three and partially filled the remaining two gaps, further revealing that these gaps involved very long tandem repeats. For example, Figure1 shows one of the gap-spanning Nanopore reads with two tandem repeats. Some of these tandem repeats have more than 1000 copies of 3–50 nt units or over 50 copies of more than 100 nt units. It is fair to say that current state-of-the-art long read assemblers are weak at reconstructing long tandem repeats.

These two applications of long-read sequencing emphasize the importance of detecting tandem repeats of more than 1000 nt from long reads. However, both commercially available long-read sequencers (PacBio and Nanopore) have high error rates, 12–20%, for raw reads (Bowden *et al.*, 2019; Weirather *et al.*, 2017; Wei and Zhang, 2018), implying that one encounters a substitution, insertion or deletion every five to eight nucleotides on average. To solve this problem, we exploit the characteristic that long tandem repeats have many occurrences of the representative unit, e.g. hundreds of copies of a nearly identical 3–200 nt unit, and we present a statistical solution to estimate the representative unit and the number of its copies from a very noisy long read.

### 1.1 Formal definitions of tandem repeats

Here, we give formal definitions for the terms relevant for describing tandem repeats, which intuitively are the repeated concatenation of a specific unit string with some level of variation amongst the instances. We start first with the case of *perfect* tandem repeats:

**Definition 1.** Let **Σ** denote a set of symbols, and $\Sigma^{+}$ be the set of non-empty strings over **Σ**. For string $u\in \Sigma^{+}$, a *perfect tandem repeat* of *u* is a concatenation of k≥1 occurrences of *u*, denoted by *u^k^*. We extend this definition by also considering $u^{k}u_{p}$, where *u_p_* is a prefix of *u*, to be a perfect tandem repeat. □

**Example 1.** Several examples of perfect tandem repeats are:

-
$\mathrm{ACGACGACG}={\left(\mathrm{ACG}\right)}^{3}$ and $\mathrm{ACGACGAC}={\left(\mathrm{ACG}\right)}^{2}\mathrm{AC}$ are perfect tandem repeats.
-
ACGACGACGACG has two decompositions, ${\left(\mathrm{ACG}\right)}^{4}$ and ${\left(\mathrm{ACGACG}\right)}^{2}$. The former representation is more informative as the latter unit has two copies of the former unit.
-
ACGACGACACGACGAC is a perfect tandem repeat with two occurrences of unit $\mathrm{ACGACGAC}={\left(\mathrm{ACG}\right)}^{2}\mathrm{AC}$, and the unit has also a shorter perfect tandem repeat ${\left(\mathrm{ACG}\right)}^{2}$.

One can enumerate all perfect tandem repeat occurrences in a string of size *n* in *O*(*n*)-time (Kolpakov and Kucherov, 1999). In reality, however, each unit occurrence of a tandem repeat typically has some low level of substitutions, insertions and deletions because the unit instances can evolve and accumulate mutations, and/or are likely to have sequencing errors. To take this into account, we define an *approximate* tandem repeat as a string that is sufficiently similar to an underlying perfect repeat:

**Definition 2.** For two strings *α*_1_ and *α*_2_ in $\Sigma^{+}$, let $\text{similarity}(\alpha_{1},\alpha_{2})$ be the length of the longest common subsequence (LCS) between *α*_1_ and *α*_2_ divided by the average of the lengths of *α*_1_ and *α*_2_. We say that *α*_1_ and *α*_2_ are *τ-similar* when for $\tau \leq 1,\;\text{similarity}(\alpha_{1},\alpha_{2})\geq \tau$. Given a threshold criterion *τ*, we call *α* an τ−*approximate tandem repeat* if and only if there exists a perfect repeat that is *τ*-similar to *α*. □

For example, consider the following approximate tandem repeat similar to perfect tandem repeat ${\left(\mathrm{ACG}\right)}^{4}$:

$$\mathrm{AC}\underline{A}\mathrm{ACGACG}\underline{G}\mathrm{CG}$$

The 3rd and 10th nucleotides are respectively substituted with A and G. So the similarity is 10/12=∼83.3%. If we set our threshold *τ* for similarity to 85%, then $\mathrm{AC}\underline{A}\mathrm{ACGACG}\underline{G}\mathrm{CG}$ would not be considered an approximate tandem repeat. However, the first nine characters, $\mathrm{AC}\underline{A}\mathrm{ACGACG}$, have a similarity of 8/9=∼88.8% to ${\left(\mathrm{ACG}\right)}^{3}$ as do the last nine letters, $\mathrm{ACGACG}\underline{G}\mathrm{CG}$. This illustrates that there can be more than one longest approximate tandem repeat of a string and that these can overlap. Moreover, these depend on the stringency *τ*, e.g. the six letters in the middle $\mathrm{ACGACG}={\left(\mathrm{ACG}\right)}^{2}$ are a perfect tandem repeat but is shorter than the above two substrings of length nine. Figure 1 shows two approximate tandem repeats. The upper left tandem repeat has a similarity of ∼87.7% with its predicted perfect tandem repeat, while the lower right repeat has a similarity of ∼69.7%.

Enumerating all approximate tandem repeats is in general intractable as the time complexity increases exponentially with the number of mutations (Domaniç and Preparata, 2007; Pellegrini *et al.*, 2010).

### 1.2 Related work

There have been a variety of heuristic methods developed for finding tandem repeats. Tandem Repeats Finder (TRF) is widely used to enumerate tandem repeats in genomes as well as in raw reads (Benson, 1999). TRF first finds candidate regions containing tandem repeats and then searches these candidate regions extensively for tandem repeats. This two-step filter-and-verify strategy has been adopted by many software programs such as ATRHunter (Wexler *et al.*, 2005), TRStalker (Pellegrini *et al.*, 2010) and TideHunter (Gao *et al.*, 2019). A number of useful heuristic algorithms have been proposed to improve the filtering step. For example, gapped q-grams (or k-mers) (Burkhardt and Kärkkäinen, 2003) are more sensitive than common ungapped q-grams, and are used by TEIRESIAS (Floratos *et al.*, 2002) and TRStalker (Pellegrini *et al.*, 2010). Fourier transform and other methods based on signal processing theory have also been employed in the literature (Brodzik, 2007; Buchner and Janjarasjitt, 2003; Gupta *et al.*, 2007; Sharma *et al.*, 2004). After the advent of next-generation short-read sequencers, efficient programs have been also developed to process numerous short reads; for example, lobSTR, TRhist and Dot2dot (Doi *et al.*, 2014; Genovese *et al.*, 2019; Gymrek *et al.*, 2012). These traditional studies were designed to detect approximate tandem repeats of relatively short length in relatively low error rate sequences.

## 2 Materials and methods

**Fig. 2.**
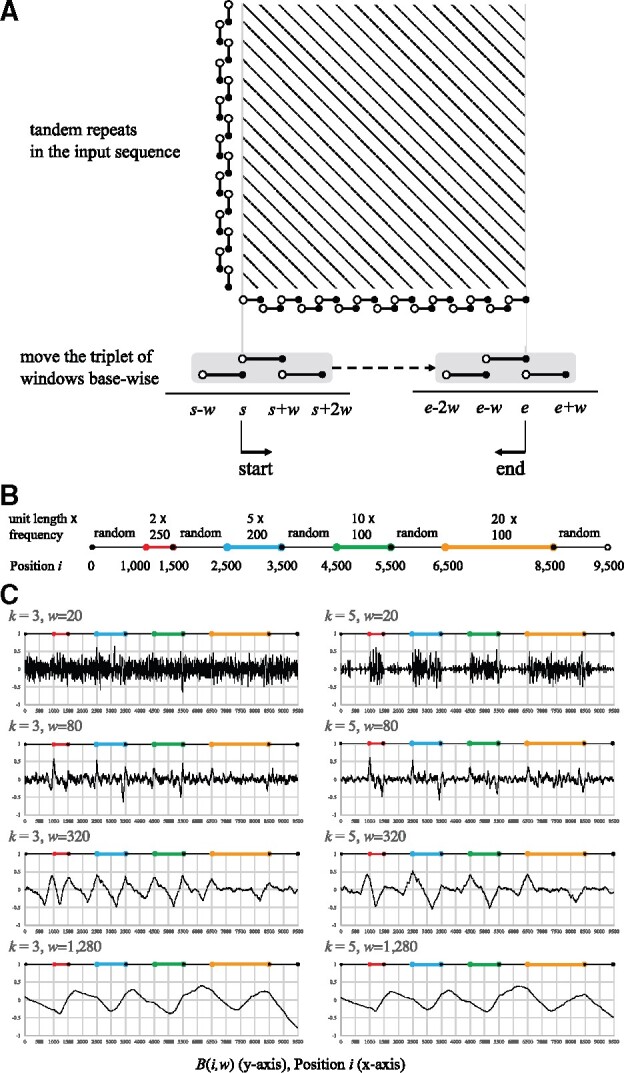
Estimating the start and end positions of tandem repeats. (**A**) A dot plot of the input sequence. Dotted lines parallel to the diagonal represent tandem repeats in the input, and the distance between neighboring dotted lines is the unit length of the tandem repeat. The boundaries of a tandem repeat are predicted by comparing *k*-mer frequency vectors of neighboring windows of size *w*. We move the triplet of windows base-wise to detect the start and end positions that optimize the formulae given in Definition 4. We may need windows of different sizes to determine the boundaries of individual tandem repeats. (**B**) Four tandem repeats (colored red, blue, green and orange) separated by random sequences in the sample input. (**C**) *B*(*i*, *w*) for k= 3 in the left column and 5 in the right, for window size *w *=* *20, 80, 320 and 1280 from the top, and for position *i*

To gain computational efficiency while retaining sensitivity, hundreds or thousands of approximate copies of the representative unit are quite informative, and we will utilize this characteristic to design an efficient and accurate program named mTR (a tool for mining Tandem Repeats). Like other filter-and-verify methods, we first find candidate regions in a noisy long read that are likely to contain a tandem repeat, and then for each candidate we estimate a unit consensus and copy number if such exist. Although the error rate of a long read is assumed to be quite high, upwards of 20%, having plenty of unit copies in a tandem repeat is informative for estimating the boundaries of the tandem repeat because a unit copy is likely to share significantly more short substrings (*k*-mers) with another copy than with other irrelevant sequences outside the tandem repeat (Fig. 2A). Using these heuristic ideas, we design novel statistical methods for predicting the boundaries of a tandem repeat and for deciding upon the most likely repeat unit.

### 2.1 Estimating tandem repeat boundaries

To approximate the start and end positions in a tandem repeat, we define several terms.

**Definition 3.** A string of length *k *>* *0 in $\Sigma^{+}$ is called a *k*-mer. When **Σ** is the set of four nucleotides {A, C, G, T}, we encode *k*-mers by mapping the nucleotides to the digits 0, 1, 2 and 3 and then viewing them as *k*-digit quaternary numbers in the range 0 to $4^{k}-1$. Given a read $R=r_{1}r_{2}\cdots r_{n}$ we consider *position i* to be the point between *r_i_* and $r_{i+1}$. So, the substring between positions *a* and *b*, R[a,b], is $r_{a+1}r_{a+2}\cdots r_{b}$ and its length is *b* – *a*. We call $W_{i}^{w}=R[i,i+w]$ the window of size *w* at position *i* and for k≤w, the *k*-mer *frequency vector* of the window is:

$${\vec{f}}_{i}^{w}=(f_{0},\ldots ,f_{j},\ldots ,f_{4^{k}-1}),$$

where *f_x_* is the number of occurrences of the *k*-mer with quaternary code *x* in the window. Finally, for two frequency vectors $\vec{f}$ and $\vec{h}$, let $\xi (\vec{f},\vec{h})\in [0,1]$ be a general normalized measure of similarity between them further defined below.

We will consider the similarity, $S_{\left(i,j\right)}^{w}$ between two windows $W_{i}^{w}$ and $W_{j}^{w}$ of the same size, to be the similarity of their respective frequency vectors. That is,

$$S_{\left(i,j\right)}^{w}=\xi ({\vec{f}}_{i}^{w},{\vec{f}}_{j}^{w}).$$

In this study, we considered using both the Pearson Correlation coefficient and the Manhattan distance metric as possible measures of similarity. Specifically, we tried $\xi (\vec{f},\vec{h})=(\rho (\vec{f},\vec{h})+1)/2\in [0,1]$ where *ρ* is the normalized Pearson correlation coefficient,

$$\rho (\vec{f},\vec{h})=\frac{\sum_{i=1}^{n}\left({\vec{f}}_{i}-\bar{f}\right)({\vec{h}}_{i}-\bar{h})}{\sqrt{\sum_{i=1}^{n}{\left({\vec{f}}_{i}-\bar{f}\right)}^{2}}\sqrt{\sum_{i=1}^{n}{\left({\vec{h}}_{i}-\bar{h}\right)}^{2}}},$$

for $n=4^{k},\;\bar{f}=\sum_{i=1}^{n}{\vec{f}}_{i}/n$ and $\bar{h}=\sum_{i=1}^{n}{\vec{h}}_{i}/n$. We also used $\xi (\vec{f},\vec{h})=1-||\vec{f}-\vec{h}|{|}_{1}/2w\in [0,1]$ where $||\vec{f}-\vec{h}|{|}_{1}$ is the Manhattan or *L*_1_ distance between $\vec{f}$ and $\vec{h}$, which is $\sum_{i=1}^{n}|{\vec{f}}_{i}-{\vec{h}}_{i}|$. □

Although the two similarity measures have different characteristics, experimental results in a later section will show that both have similar sensitivities for prediction of tandem repeats (see Fig. 4).

**Example 2.** Let *w *=* *4, *k *=* *1 and R=TCAGACACACACGGTC. Consider the following four non-overlapping windows of size *w*, and their *k*-mer frequency vectors:

**Table:** 

$W_{0}^{4}$	=R[0,4]	=TCAG	${\vec{f}}_{0}^{4}$	**=** (1,1,1,1)
$W_{4}^{4}$	=R[4,8]	=ACAC	${\vec{f}}_{4}^{4}$	= (2,2,0,0)
$W_{8}^{4}$	=R[8,12]	=ACAC	${\vec{f}}_{8}^{4}$	= (2,2,0,0)
$W_{12}^{4}$	=R[12,16]	=GGTC	${\vec{f}}_{12}^{4}$	= (0,1,2,1)

As we set *k *=* *1, the respective elements in a vector show the frequencies of 1-mers A, C, G and T. Among all pairs of neighboring windows, the frequency vectors of the second and third windows, ${\vec{f}}_{4}^{4}$ and ${\vec{f}}_{8}^{4}$, are the most similar in terms of the Manhattan similarity:

$$S_{\left(8,12\right)}^{4}(=0.25)<S_{\left(0,4\right)}^{4}(=0.5)<S_{\left(4,8\right)}^{4}(=1)\;\;$$

The idea of *k*-mer frequencies has been widely used in string processing for over a quarter of century. For example, Esko Ukkonen used them to lower bound edit distance for approximate search (Ukkonen, 1992). Computing ${\vec{f}}_{i}^{w}$ for i∈[0,|R|−w] can be performed in time linear in the length of *R* because the frequency vector of the next window, ${\vec{f}}_{i+1}^{w}$, can be computed incrementally from ${\vec{f}}_{i}^{w}$ in a constant time. Precisely, in ${\vec{f}}_{i}^{w}$, decrement the frequency of *k*-mer R[i,i+k] and increment that of *k*-mer R[(i+1+w)−k,i+1+w].

The similarity between two windows can be greatly affected by the error rate of the data if the underlying value of *k* is not chosen carefully. Specifically, the larger *k*, the more likely an error will ‘knock out’ *k*-mers that would correlate if the data were perfect, resulting in a rapid degradation of the correlation score between windows that would highly correlate in the case of perfect data. Let *ϵ* denote a sequencing error rate. With the simplified assumption that sequencing errors are independent and identically distributed with a probability of *ϵ*, the probability that a *k*-mer has no sequencing errors is ${\left(1-\epsilon \right)}^{k}$. The typical average error rate of long-read sequencing ranges from 11 to 20%, so we will assume that ϵ=20% in order to capture the worst case scenario. For k=1,…,6, the probability ${\left(1-\epsilon \right)}^{k}$ is 80, 64, 51.2, 41.0, 32.8 and 26.2%. So for long read data, we find it desirable to use short *k*-mers of length 3, 4 or 5 for better detection of tandem repeats, and to use longer windows with more *k*-mer occurrences to further reduce the effect of sequencing error.

We now describe how to estimate the boundaries of a tandem repeat. Suppose that position *s* is at the start boundary of a tandem repeat. Window $W_{s}^{w}$ at the start is likely to be more similar to the next non-overlapping window $W_{s+w}^{w}$ than to its previous window $W_{s-w}^{w}$ if the window size *w* is longer than the tandem repeat unit and if the tandem repeat spans more than 2*w* nucleotides, both conditions one expects to meet empirically for some choice of *w* (Fig. 2A). So, we defin*e*

$$B(i,w)=S_{\left(i,i+w\right)}^{w}-S_{\left(i-w,i\right)}^{w},$$

which is the difference between the correlations of the two sequential windows to the right of position *i* and the two windows separated by position *i*. We expect this value to peak at the start of the left boundary of a tandem repeat. Symmetrically, let

$$E(i,w)=S_{\left(i-2w,i-w\right)}^{w}-S_{\left(i-w,i\right)}^{w}=-B(i-w,w),$$

which is the difference between the two sequential windows to the left of position *i* and the two windows separated by position *i*, whose value we expect to peak at the end or right boundary of a tandem repeat. So given a choice of window and *k*-mer sizes, *w* and *k*, we define the start- and end points of a putative tandem repeat as follows:

**Definition 4.** The start and end points of a putative tandem repeat for *w*, denoted by *b*(*w*) and *e*(*w*), are required to meet:

- *b*(*w*) locally maximizes *B*(*j*, *w*) within distance *w* of it, i.e. B(j,w)≤B(b(w),w) for |j−b(w)|≤w.
- *e*(*w*) is the closest point to *b*(*w*) such that b(w)+w≤e(w), and *e*(*w*) locally maximizes *E*(*j*, *w*) within distance *w* of it (i.e. E(j,w)≤E(e(w),w) for |j−e(w)|≤w).

Computing these values for a given *w* and *k* takes O(|R|) time as it suffices to compute *B*(*i*, *w*) and *E*(*i*, *w*) at every value of *i* and this is done easily if one precomputes ${\vec{f}}_{i}^{w}$ for i=0,…,|R|−w also in O(|R|) beforehand. We will show shortly that the start and end positions returned depend significantly on *w* and to a lesser extent *k*, so to utilize this approach, a good choice of *w* is needed.

It remains to select ‘good’ choices of *w* and *k* as a function of the unit-size and underlying error rate. Consider as an example the situation in Figure 2B where *R* has four tandem repeat types with different unit lengths and frequencies, and the read itself has 10% mismatch, 5% insertion and 5% deletion rates. Figure 2C shows the distributions of *B*(*i*, *w*) for k= 3 and 5, and for w= 20, 80, 320 and 1280, showing how *B*(*i*, *w*) depends on the values of *k* and *w*. When *w *=* *20 in the top row, the distributions are too noisy to detect peaks at the boundaries of any of the tandem repeats. Peaks at the starts become evident when w= 80 and 320 because windows have plenty of 20-nt unit occurrences. When w=1280, however, peaks at the starts of the red, blue and green tandem repeat disappear because 1280-nt windows are longer than the tandem repeats.

Conceptually, one can see that we seek a value of *w*, say w*, such that B(b(w*),w*) is maximal. Ideally one would try all values of *w* but this would require excessive amount of calculation. Instead, we sample a geometric progression of *w*, such as $w=a2^{l}$ for l=0,1,… and for some starting value *a*. Here, we explain the rationale to support this sampling. To detect a tandem repeat of length *L* with unit *u*, it is important to meet the following conditions for positive constant c (<1):

$$2|u|\leq w\leq L/2\;\;\text{and}\;\;k\leq |u|<c\times 4^{k}.$$

The first condition implies that windows of size *w* contain two or more occurrences of unit *u*, and the entire repeat has at least two windows of size *w*. The second condition demands that the unit size is greater than or equal to *k*, and the number of different *k*-mers in a tandem repeat of *u*, which is ≤|u|, is also smaller than $c\times 4^{k}$, to characterize the unit in terms of a relatively small subset of *k*-mers for say *c *=* *1/4. To accommodate varying error rates, we sample *k *=* *5 for repeats with a large span, whereas we sample *k *=* *1, 3 for potentially small repeats. In a typical setting when |u|≤500 and $L<10^{5}$, these requirements are met by one of twenty patterns, $\left(k,w)=(5,5\times 2^{l}\right)$ for l=0,1,…,11, $\left(k,w)=(3,5\times 2^{l}\right)$ for l=0,1,2,3,4 and $\left(k,w)=(1,5\times 2^{l}\right)$ for l=0,1,2.

### 2.2 Assembling *k*-mers into tandem repeat units

After predicting candidate tandem repeat ranges for various values of *w* and *k*, for each range [s,e] we attempt to identify the consensus repeat unit that in tandem spans the range. To do so we utilize the characteristic that there are enough copies of the unit in tandem so that many short *k*-mers of this unit will be preserved even if the error rate is ∼20%. The de Bruijn graph of *k*-mers for some value of *k* is a widely accepted approach for assembling a genome from accurate short reads (Compeau *et al.*, 2011; Pevzner *et al.*, 2001; Zerbino and Birney, 2008) and has also been used for long read error correction (Tischler and Myers, 2017). For an auspicious value of *k*, frequent *k*-mers will be parts of exact copies of the unit, while infrequent *k*-mers will contain sequencing errors. Thus, conceptually we seek the heaviest cycle in a *k*-mer de Bruijn graph over R[s,e] for an appropriate value of *k*.

**Fig. 3.**
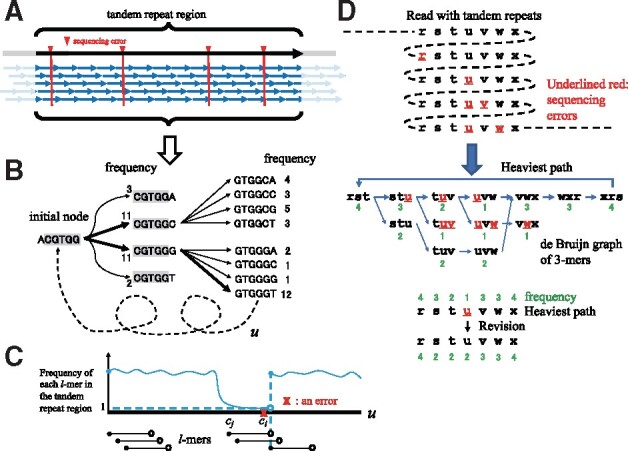
Estimating the unit of a hidden tandem repeat in a noisy long read. (**A**) Select a value for *k* such that many short *k*-mers have no sequencing errors in an approximate range of a hidden tandem repeat, allowing us to reconstruct the unit string of the tandem repeat based on the de Bruijn graph approach. (**B**) Searching the de Bruijn graph of *k*-mers (e.g. *l *=* *6) in raw reads to assemble *k*-mers into the original tandem repeat unit. Our greedy algorithm selects the next node of the maximum frequency until reaching the initial node and outputs the best path (denoted by *u*). (**C**) Frequency of each *k*-mer of *u* in the focal range. The frequency of a *k*-mer becomes much lower than the average frequency when it has an error. (**D**) This example illustrates how the consensus sequence with a *k*-mer of frequency one is fixed by our algorithm. In the top, a read with tandem repeats is shown. Red underlined characters represent sequencing errors that are either substitutions, deletions or insertions. In the middle, the de Bruijn graph of 3-mers in the input read shows the heaviest path (bold line) that our greedy algorithm outputs. The algorithm moves from the most frequent node rst to stu with an error of frequency 3 rather than to the error-free stu of frequency 2, thereby going through the three consecutive nodes with errors. Our algorithm revises the consensus by scanning it forward and backward and fixes the sequencing error *u* colored red. Although the tandem repeat unit is quite short in this illustrating example, such erroneous patterns are typical of long tandem units >50 nt in size, and our greedy algorithm is effective in reducing such errors

Proceeding more formally, there is an edge $v\to_{a}z$ from *k*-mer *v* to *k*-mer *z* if *v*=*bx* and *z*=*xa* for some a,b∈Σ and k−1-mer *x*. Given a set of vertices *V*, let *next*(*V*)={*z* : $v\to_{a}z$ for some v∈V, a∈Σ and *z* has maximum frequency }. Note that most of the time next({v}) is a singleton set as ties for the heaviest successor are rare. Let ${\text{next}}^{h}(V)=\text{next}({\text{next}}^{h-1}(V))$ for *h *>* *1. With these preliminaries, our heuristic algorithm for finding a heavy cycle is as follows:

**Procedure 1.** For each *k* (say, k=2,…,15), find a cycle in the *k*-mer de Bruijn graph as follows (see Fig. 3A and B):

1. Count the frequency of each *k*-mer in the range R[s,e], select the most frequent *k*-mer *κ* as the start vertex in the underlying de Bruijn graph, and initialize the consensus unit string *u* to the empty string.
2. If the search is at vertex *v*, compute successive values of ${\text{next}}^{h}(\{v\})$ until its cardinality is 0 or 1, or *κ* is in the set. If 0, the search fails. Otherwise, let *z* = ${\text{next}}^{h}(\{v\})$ if it is a singleton, or *κ* if it contains it. Traverse the *h* edges from *v* to *z* concatenating their labels to *u* and advance *v* to *z*.
3. Repeat the above step until the initial *k*-mer *κ* is reached. The unit string *u* is now a potential consensus repeat unit for the repeat.

If there is more than one most frequent *k*-mer, then each is examined as the start vertex and the heaviest weight cycle that maximizes the sum of the frequencies of the *k*-mers in the cycle is taken if more than one search is successful. From the start vertex, we also search the *k*-mer de Bruijn graph backward, traversing an edge $v\leftarrow_{a}z$ from *z*=*xa* to *v*=*bx* for some a,b∈Σ and k−1-mer *x*. If the search fails at some point because there is no move available, then no unit is produced for the given value of *k*. If the procedure fails for all values of *k* attempted, then the proposed repeat range is ignored. As the last step, for each search with a given value of *k* that succeeded in producing a cycle *u*, *u* is aligned to R[s,e] using wraparound dynamic programing (Fischetti *et al.*, 1993; Miller and Myers, 1988), to calculate σ(u,R[s,e]) which is the maximum of the number of matches minus the number of differences over all possible alignments between a tandem repeat of *u* and R[s,e], i.e. (e−s)−δ(u,R[s,e]) where *δ* is Levenshtein distance. If multiple candidate units *j* are obtained for different values of *k*, we select the one that has the best alignment score *σ*. □

The above procedure may use a very low-frequency *k*-mer in order to be able to find a cycle for *u*. For the case where the frequency of the unit is quite low (e.g. 10 or less), it could be that the low-frequency *k*-mers are correct, but in other instances they may indicate an error in the consensus for *u* as the difference in frequency between *k*-mers with and without error is typically significantly large for the case of high copy number tandem repeats. Most often the difference is a single substitution, insertion or deletion. A typical observation is that, when scanning the *k*-mers along the cycle spelling *u* from the beginning to end, the *k*-mer frequency is low when the *k*-mer has an error, but rises sharply toward the average frequency of *k*-mers in *u* when the *k*-mer becomes free of error (Fig. 3C). So we search the path spelling *u* for such transition positions indicating errors by scanning it both forward and backward. Let *c_j_* denote the nucleotide at position *j*. After locating a position *i* with a potential error, we modify *k*-mers having *c_i_* of the form $c_{j}\ldots c_{i}\ldots c_{j+l-1}$ by substituting *c_i_* with other nucleotides, by deleting *c_i_* or by inserting another nucleotide after *c_i_*:

**Table:** 

No operation	$c_{j}\ldots c_{i-1}c_{i}c_{i+1}\ldots c_{j+l-1}$	
Substitutions	$c_{j}\ldots c_{i-1}a\;c_{i+1}\ldots c_{j+l-1}$	(a∈Σ and $a\neq c_{i}$)
Deletion	$c_{j-1}\ldots c_{i-1}\;\;\;c_{i+1}\ldots c_{j+l-1}$	
Insertions	$c_{j}\ldots c_{i-1}c_{i}a\;c_{i+1}\ldots c_{j+l-1}$	(a∈Σ)

To fix the error, from one of the above substitutions, deletions and insertions, we select the operation best able to maximize the sum of the frequencies of all *k*-mers in the operation for j=i−l+1,…,i.

After correcting some errors in *u*, we try to remove additional errors using the traditional multiple alignment approach. We align R[s,e] to *u* using the wraparound dynamic programming algorithm, derive a multiple alignment of unit occurrences with errors, examine each column of the multiple alignment, and revise *u* if needed. In detail, let *j* be a position in *u*, and let $n_{j}^{a}$, *del_j_* and $ins_{j}^{a}$, respectively, denote the number of a∈Σ aligned at position *j*, the number of times that *c_j_* is deleted and the number of a∈Σ inserted after *j*. We consider replacement of *c_j_* with another $a(\neq c_{j})$, deletion of *c_j_* and insertion of *a* if $n_{j}^{a}$, *del_j_*, $ins_{j}^{a}$ are significantly high. To examine the significance, we set *ϵ* to the average error rate in the alignment, and make the assumption that *c_j_* is replaced with another nucleotide or deleted at the identical probability ϵ/4, and that a∈Σ is inserted after position *j* at the probability ϵ/4. The significance is also dependent on the number (or, depth) of units in the multiple alignment, and let *d* denote the depth. When we observe *K* replacements of *c_j_* with *a* (deletions of *c_j_*, insertions of *a*, respectively), let *p*(*K*) denote the probability that $n_{j}^{a}$ (*del_j_*, $ins_{j}^{a}$) is *K* or more. Thus, we have:

$$p(K)=\Sigma_{k=K}^{d}{\;}_{d}C_{k}{\left(1-\epsilon /4\right)}^{d-k}{\left(\epsilon /4\right)}^{k}.$$

Let |u| denote the length of *u*; thus, we have 8×|u| hypotheses that substitutions, deletions and insertions are required to fix errors in *u*. It is not necessary to correct *u* if it is the underlying unit, but it may be necessary to correct a small number of errors in *u* if *u* is nearly identical to the true unit. To determine whether *u* needs correction, we test the null hypothesis that none of the 8×|u| hypotheses is true by checking p(K)≤1%/(8×|u|) for each hypothesis according to the Bonferroni correction. We perform a correction when *p*(*K*) exceeds the significance level 1%/(8×|u|).

### 2.3 Computing non-overlapping multiple tandem repeats

After applying the procedures described in Sections 2.1 and 2.2, we effectively have a collection of, say *n*, candidate tandem repeats ${\left\{T_{i}\right\}}_{i=1}^{n}$ where $T_{i}=(s_{i},e_{i},u_{i},\sigma_{i}$**)** designating that *u_i_* matches $R[s_{i},e_{i}]$ with alignment score of *σ_i_* = $\sigma (u_{i},R[s_{i},e_{i}]$**)** as defined earlier. Often these candidates overlap, so the final task is to find a subset of these candidates and if necessary subranges of some, so that the resulting disjoint tandem models reasonably explain all the tandem repeats in *R*. In the disjoint version of the problem we say that *T_i_* can be chained to *T_j_* if and only if $e_{i}\leq s_{j}$, written $T_{i}\to T_{j}$. The problem is then effectively that of finding a chain of maximal weight. Computing an optimal, *disjoint* chain that maximizes the chain weight is solvable in O(n log n) time, where *n* is the number of candidates (Myers and Miller, 1995).

A complexity occurs when *T_i_* and *T_j_* overlap by some amount, typically small, i.e. $s_{i}<s_{j}<e_{i}<e_{j}$ where $e_{i}-s_{j}$ is not too large. In this case, we would like to pick a point *x* at which *T_i_* should end and *T_j_* begin. Let the score of splitting the overlap at *x* be:

$$o(x)=\sigma (u_{i},R[s_{i},x])+\sigma (u_{j},R[x,e_{j}])$$

and choose the split point $x^{*}$ as the value of $x\in [s_{j},e_{i}]$ that maximizes *o*(*x*). We can compute *o*(*x*) in $O(|e_{i}-s_{j}|(|u_{i}|+|u_{j}|)$ by storing $\sigma (u_{i},R[s_{i},x])$ for $x=s_{i},\ldots ,e_{i}$ in the initial computation of $\sigma (u_{i},R[s_{i},e_{i}])$ described in Section 2.2 for each candidate, and similarly $\sigma (u_{j},R[x,e_{j}])$ for $x=s_{j},\ldots ,e_{j}$

We could then go on to pose a *non-disjoint* chaining model, where *T_i_* can be chained to *T_j_* at weight $\sigma^{*}$ if and only if *s_i_* < *s_j_* and $\sigma^{*}$ = $\sigma_{j}-\sigma (u_{i},R[x^{*},e_{i}])$ where $x^{*}=e_{i}$ when *T_i_* and *T_j_* do not overlap ($e_{i}\leq s_{j}$), written $T_{i}\to_{\sigma^{*}}T_{j}$. A chain in this construction has weight equal to the sum of each unit aligned to the intervals defined by the sequence of optimal division points between the candidates in the chain. Unfortunately, it comes at the cost of $O(n^{2})$ time to compute.

To retain an efficient algorithm, we instead heuristically solve a ‘pseudo-disjoint’ chaining problem, by considering *T_i_* and *T_j_* to be disjoint if their overlaps is less than some small limit *l*, say 10 nt. Thus we retain O(n log n) performance. Then if the optimal chain produced contains a pair of *T*’s that overlap slightly we choose the division point between them as above to produce a truly disjoint list of tandem repeats and their consensus units.

## 3 Experimental results

### 3.1 Synthetic datasets for performance analysis

To measure the computational performance of our program, we first generated synthetic datasets with 1000 strings such that each string had an approximate tandem repeat of a single unit within it. We generated the tandem repeats in each dataset by setting the following three parameters to representative values:

- The unit length is one of 2, 5, 10, 20, 50, 100 or 200.
- The frequency of the unit is one of 10, 20, 50, 100 or 200.
- We generated synthetic long reads using Badread, a program widely used for simulation PacBio and Nanopore reads with default settings (Wick, 2019).

Before and after each tandem repeat, we inserted random strings of the same length as the tandem repeat, to examine whether the program was able to predict the boundary of the focal tandem repeat correctly. The synthetic datasets and programs for evaluating the sensitivity of processing the data are available at https://github.com/morisUtokyo/mTR.

### 3.2 Comparison with TRF and TideHunter

Using the synthetic benchmark datasets, we compared the sensitivity and computational performance of our program, mTR and TRF (Benson, 1999). We selected TRF for comparison for several reasons. First, TRF has been maintained and updated for about 20 years (since 1999). Second, it can handle long reads with long tandem repeats in a reasonable amount of time. Lastly, it is the most widely used software program to detect tandem repeat expansions. We used the latest version of TRF (version 4.09). We also performed a comparison using TideHunter, which is capable of handling long reads (Gao *et al.*, 2019). We did not evaluate tools designed for *small* tandem repeats such as TEIRESIAS (Floratos *et al.*, 2002), ATRHunter (Wexler *et al.*, 2005), TRStalker (Pellegrini *et al.*, 2010), lobSTR (Gymrek *et al.*, 2012), TRhist (Doi *et al.*, 2014) and Dot2dot (Genovese *et al.*, 2019), as we seek tandem repeats in excess of 1000 nt. To empirically measure performance we used a server with an Intel(R) Xeon(R) CPU E5-2680 v3 with a clock rate of 2.5 GHz, and GCC (version 4.9.3) to compile the source codes. In passing we note that mTR requires less than 100 MB of main memory, even when the input read is 1 million nt in size and thus can actually be run on conventional laptops.

**Fig. 4.**
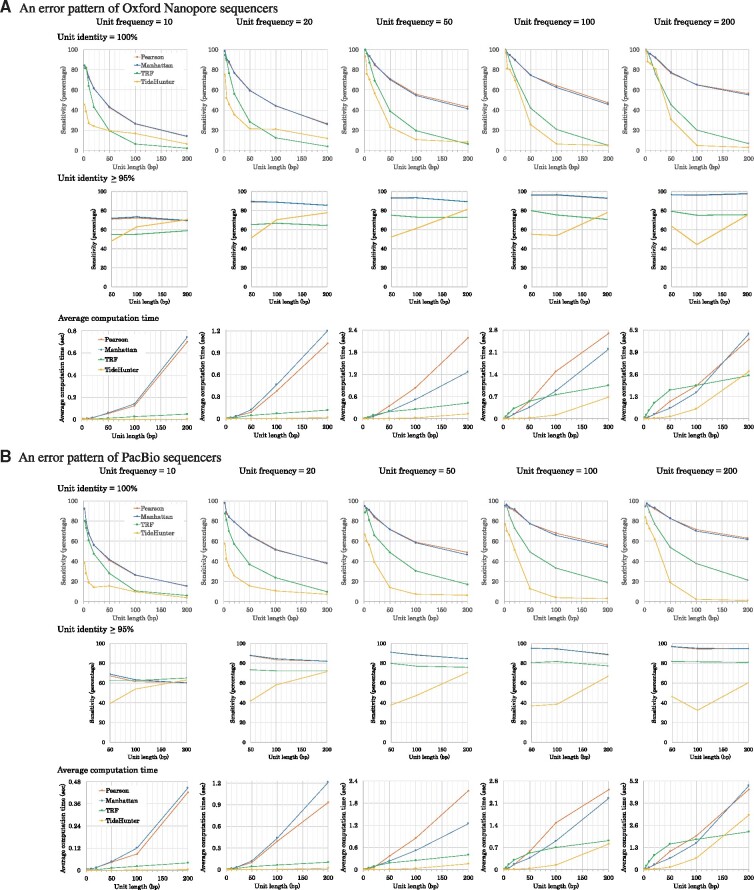
Sensitivity and computational performance of mTR, TRF and TideHunter, as applied to two types of datasets with different error patterns (**A** and **B**). The first had an error pattern typical of Nanopore sequencers, and the second dataset had an error pattern typical of PacBio sequencers. The graphs show the experimental results obtained by mTR when using Manhattan similarity (blue) and Pearson’s correlation coefficient (red), and those obtained by TRF (green) and TideHunter (orange). The frequency of units in each synthetic dataset is shown above each graph. In each graph, the x-axis shows the unit length. The y-axes in the top and middle graphs of A and B show the sensitivity, while the y-axes in the bottom graphs show the average computation time. The top graphs of A and B show the sensitivity when the predicted and true units match at identity 100%, while the middle graphs match at identity ≥95%. The middle graphs do not show sensitivity for unit length ranging from 2 to 10 because the unit identity of 100% and that of ≥95% are equivalent in this range

To determine the sensitivity, each approximate tandem repeat was associated with its original perfect tandem repeat and unit string. Due to the high error rates, it is difficult to accurately predict the frequency of a unit in an approximate tandem repeat. However, estimating the consensus unit string is more feasible. Therefore, we assessed the ability of the programs to perfectly predict the unit string in each synthetic read. Figure 4 plots the sensitivity and computational performance of mTR and TRF as a function of unit length for a number of choices of unit frequency. We applied TR and mTR with Manhattan similarity and Pearson’s correlation coefficient to the synthetic datasets. Overall, mTR outperformed TRF in terms of sensitivity on all trials. The two similarity measures of mTR were comparable in terms of sensitivity and computational efficiency in most cases. Both mTR and TRF are in principle linear in the span of the repeat interval (unit length x unit frequency), but mTR appears to have a higher constant term and a smaller linear term due to the number of parameter ranges explored in both the detection and consensus phases offset by the simplicity of the linear computation for each parameter setting. This implies that mTR is slower than TRF for small tandem repeats and vice versa for large ones as seen in Figure 4. Not seen in this figure, is the fact that TRF stops searching earlier more often than mTR for large tandem repeats, implying that in fact mTR is doing more work actually finding and listing large tandem repeats.

The top graphs in Figure 4A and B show the sensitivity when the estimated and true units match at identity 100%. It is also informative to know how many of predicted units approximate the true units if we relax the sensitivity of the unit. The middle graphs in Figure 4A and B show the sensitivity when the identity is 95% or more, i.e. allowing at most two, five and ten errors in units of length 50 nt, 100 nt and 200 nt, respectively. Under this relaxed condition, mTR still outperformed TRF in most cases except in a few where the unit frequency was 10 and the identity was 95% or more (see Fig. 4B). Essentially, when the unit frequency is sufficiently large, mTR is capable of producing repeat units nearly identical to the true underlying units.

Although the Pearson correlation coefficient and Manhattan similarities, have different characteristics, Figure 4 shows that their sensitivities for prediction of the true representative unit are similar and it is difficult to state which has the best computational performance.

We also examined highly accurate reads. In this setting of a low error rate, no significant difference between mTR and TRF was observed because tandem repeats were easily detected. Specifically, we randomly generated 1000 synthetic reads for 65 patterns at an error rate of 2% (respective substitution, deletion and insertion rates were 1, 0.5 and 0.5%), the unit length was one of 2, 3, 4, 5, 6, 7, 8, 9, 10, 20, 50, 100 or 200; and the frequency of the unit was one of 10, 20, 50, 100 or 200. In all the 65 cases, both mTR and TRF output results of ≥99% sensitivity, mTR was the winner in 22 cases, TRF won in 14 cases and mTR and TRF were tied in 29 cases.

**Fig. 5.**
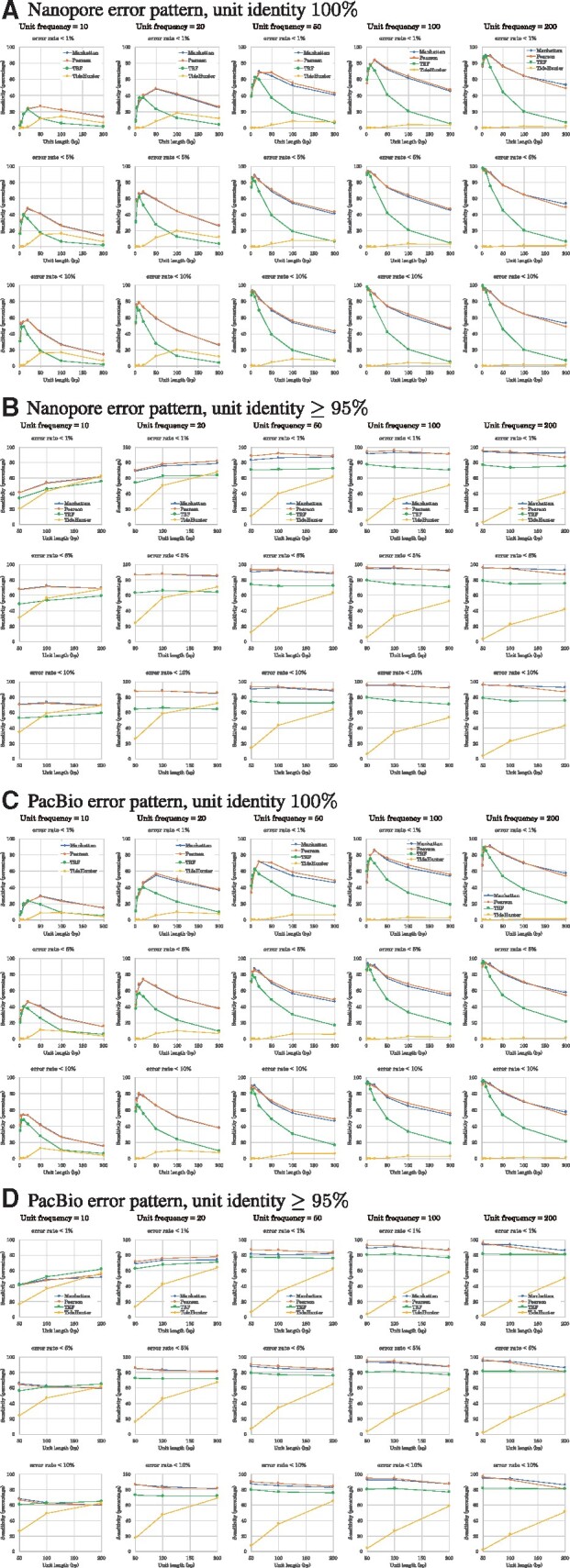
Sensitivity of mTR (Manhattan similarity, blue; Pearson’s correlation coefficient, red), TRF (green) and TideHunter (orange), as applied to two types of datasets with different error patterns typical of Nanopore (**A, B**) and PacBio sequencers (**C, D**). In each graph, the x-axis shows the unit length, and the y-axis shows the sensitivity when the predicted and true units match at identity 100% (A, C) or 95% (B, D) and the error rate between the true and predicted tandem repeat lengths is bounded by 1, 5 or 10%

We then investigated how much portion of the true tandem repeat was covered by a predicted tandem repeat. Suppose that tandem repeat TR_pred_ is predicted for a given tandem repeat TR_true_ in a noisy long read, let $\text{len}({\text{TR}}_{\text{pred}})$ and $\text{len}({\text{TR}}_{\text{true}})$ be their lengths, and let $\left|\text{len}({\text{TR}}_{\text{pred}})-\text{len}({\text{TR}}_{\text{true}})\right|$ denote the difference of the two lengths. The error rate of predicting the tandem repeat length is defined as $\frac{\left|\text{len}({\text{TR}}_{\text{pred}})-\text{len}({\text{TR}}_{\text{true}})\right|}{\text{len}({\text{TR}}_{\text{true}})}.$ We consider the conditions when the match rate between the units of TR_pred_ and TR_true_ is either 100% or >95%, and the error rate of predicting tandem repeat length is bounded by 1, 5 or 10%. We evaluated the sensitivity of individual tools to predict tandem repeats that met each of the above conditions using synthetic Nanopore and PacBio reads (Fig. 5), and found that mTR outperformed TRF in most cases except for PacBio synthetic reads when the unit frequency was 10.

### 3.3 Finding neighboring multiple tandem repeats

We also assessed whether mTR could detect multiple adjacent tandem repeats using synthetic and in the next subsection on real data. Determining the sensitivity and efficiency of mTR when applied to all possible synthetic datasets is intractable, because of the exponential number of combinations of neighboring tandem repeats, differing in units and unit frequencies. We simply generated five typical tandem repeats according to the parameter values given in Table 1. These are all detectable with a probability of >98% under the Nanopore error pattern. We tested all 10 combinations of pairs of these 5 tandems and confirmed that mTR could detect both for all pairs.

**Table 1.** Parameter values for generating the units of five tandem repeats

Size	3	5	10	20	50
Frequency	50	50	100	200	200

### 3.4 Applications to real examples

We have been using mTR to enumerate tandem repeats from PacBio and Nanopore raw reads collected from individual human genome data (Ishiura *et al.*, 2018) and *C.elegans* (Yoshimura *et al.*, 2019). We applied mTR and TRF to real PacBio long reads filled with tandem repeats of typical higher order repeats (HOR) in human chromosome X. These reads were collected from CHM13 (haploid sample) using PacBio CLR and CCS (HiFi) modes (Vollger *et al.*, 2020). The HOR unit consists of 12 occurrences of a ∼171-bp alpha satellite and is ∼2 kbp in size. Both mTR and TRF could detect tandem repeats of the ∼171-bp alpha satellite; however, neither was able to capture the tandem repeats of HORs.

We examined whether mTR could detect well-known tandem repeat expansions, such as the (CGG) repeats in the 5′UTR region of FMR1 (in human chromosome X) and the (CAG) repeats in the first exon of HTT (in human chromosome 4) from real PacBio continuous long reads (CLR) collected from HG002, which is a diploid sample (Aaron Wenger *et al.*, 2019). CLRs were used in place of CCS/HiFi reads because CLRs contain many more sequencing errors, compared with CCS/HiFi reads. mTR could detect both repeats from the raw reads. In the case of the (CGG) repeat in FMR1, the median repeat length in 19 reads was 170 bp, which was consistent with the 169 bp length in the reference hg38. For the (CAG) repeat in HTT, two repeat lengths were observed, ∼80 and ∼60 bp in 41 reads, which would represent two individual haplotypes.

**Fig. 6.**
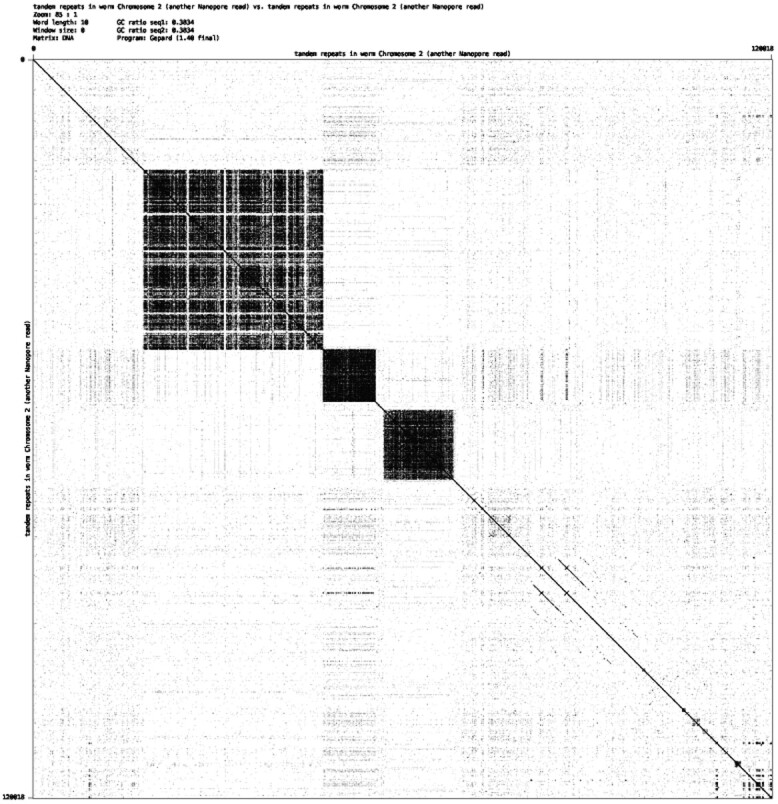
A self-dot plot of a ∼120k-nt Nanopore read with three different types of neighboring long tandem repeats. The upper left tandem repeat has 1123 copies of a 27-nt unit and matches its corresponding perfect tandem repeat at an identity of ∼73.5%; the middle tandem repeat has 53 copies of a 159-nt unit and matches at ∼89.9%; and the lower right tandem repeat has 63 copies of 183-nt unit and matches at 90.6%

Figure 6 shows another Nanopore read, ∼120k nt in length, which has three large tandem repeats. The longest tandem repeat has 1123 copies of a 27-nt unit and matches its corresponding perfect tandem repeat at an identity of ∼73.5%, which is fairly low due to several long insertions into the tandem repeat. In general, mTR assumes the presence of SNVs and short or long insertions/deletions in a tandem repeat that can be shown and examined in the alignment between the input sequence and the tandem repeats of the representative unit. Although we assumed that both of the long-read sequencing technologies (PacBio and Nanopore) have an error rate of 12%∼20%, we often observe long approximate tandem repeats that match their unit model at an identity of only ∼70% (Yoshimura *et al.*, 2019). This is because the unit copies in a long tandem often vary slightly from one another. Therefore, to identify tandem repeats from noisy reads, one must allow an error rate greater that of the read itself in order to accommodate the natural variation in the unit across the length of the tandem repeat.

## 4 Conclusion and discussion

Although long tandem repeats of more than 1000 nt have been largely unexplored, long-read sequencing technologies make it possible to sequence the entire length of many tandem repeats. As long-read sequencing technologies, such as PacBio and Nanopore, suffer from high sequencing error rates, we developed an efficient method for extracting tandem repeats in the face of such high error. Our method is based on leveraging *k*-mers short enough to occur error free with sufficient frequency to be detected and then using the frequency profile vectors of the *k*-mers to detect potential tandem repeat boundaries. We further used a de Bruijn graph of the *k*-mers in a candidate interval to reconstruct the representative unit. The experimental results demonstrated that our program, mTR, outperformed TRF, a widely used program for detecting tandem repeats, in terms of sensitivity. Our algorithm aligns the representative unit to the input sequence using wraparound dynamic programming and estimates the repeat boundaries. This dynamic programming may shift the repeat boundaries by at a maximum of one repeat unit.

As revealed in Figure 4, mTR’s performance weakens as the number of units decreases and their length increases. Primarily this is due to the unit consensus portion of our method failing to find a long cycle of short *k*-mers that occur with low frequency. This problem is relevant to genome science because many unresolved regions in genomes are known to be filled with such ‘macro’ tandem repeats, including the centromeres and the histone and rRNA clusters. A future line of research is to explore other methods specifically tuned to solve for the case of these macro-tandem repeats.

Another research target is the reuse of tandem repeat information in noisy long reads for genome assembly. Traditional genome assembly strategies ignore tandem repeats (by masking them), to avoid any effect thereof when overlapping different reads into contigs. Although most of the nucleotide differences in tandem repeats are unlikely to be informative in overlapping raw reads, some nucleotide patterns contained therein may be unique and can serve as markers when assembling tandem repeats.

## Funding

This work was supported in part by the Grant-in-Aid for Scientific Research on Innovative Areas [16H06279] (S.M.) and the Japan Agency for Medical Research and Development (GRIFIN) (S.M.).

*Conflict of Interest*: none declared.

## Data availability

Programs for generating the synthetic datasets and for evaluating the sensitivity of processing the data are available at https://github.com/morisUtokyo/mTR.
